# Diminished DEFA6 Expression in Paneth Cells Is Associated with Necrotizing Enterocolitis

**DOI:** 10.1155/2018/7345426

**Published:** 2018-10-21

**Authors:** Laszlo Markasz, Alkwin Wanders, Laszlo Szekely, Helene Engstrand Lilja

**Affiliations:** ^1^Department of Women's and Children's Health, Uppsala University, Uppsala, Sweden; ^2^Department of Biomedical Sciences, Umeå University, Umeå, Sweden; ^3^Department of Laboratory Medicine, Division of Pathology, Karolinska Institute, Stockholm, Sweden

## Abstract

**Background:**

Necrotizing enterocolitis (NEC) is the most common gastrointestinal disorder in premature infants with a high morbidity and mortality. Paneth cell dysfunction has been suggested to be involved in the pathogenesis of NEC. Defensin alpha-6 (DEFA6) is a specific marker for Paneth cells acting as part of the innate immunity in the human intestines. The aim of this study was to investigate the expression of DEFA6 in infants with NEC.

**Materials and Methods:**

Infants who underwent bowel resection for NEC at level III NICU in Sweden between August 2004 and September 2013 were eligible for the study. Macroscopically vital tissues were selected for histopathological evaluation. All infants in the control group underwent laparotomy and had ileostomy due to dysmotility, and samples were taken from the site of the stoma. DEFA6 expression was studied by immunohistochemistry. Digital image analysis was used for an objective and precise description of the samples.

**Results:**

A total of 12 infants were included in the study, eight with NEC and four controls. The tissue samples were taken from the colon (*n* = 1), jejunum (*n* = 1), and ileum (*n* = 10). Both the NEC and control groups consisted of extremely premature and term infants (control group: 25–40 gestational weeks, NEC group: 23–39 gestational weeks). The postnatal age at the time of surgery varied in both groups (control group: 4–47 days, NEC group: 4–50 days). DEFA6 expression in the NEC group was significantly lower than that in the control group and did not correlate with gestational age.

**Conclusion:**

The diminished DEFA6 expression in Paneth cells associated with NEC in this study supports the hypothesis that alpha-defensins are involved in the pathophysiology of NEC. Future studies are needed to elucidate the role of alpha-defensins in NEC aiming at finding preventive and therapeutic strategies against NEC.

## 1. Introduction

Necrotizing enterocolitis (NEC) is a serious gastrointestinal disorder that affects 10–15 percent of premature infants with a birth weight under 1500 g [1]. The mortality rates are up to 50% for infants requiring surgery [2]. Morbidity includes poor neurodevelopmental outcome and short bowel syndrome [3].

Current evidence suggests a multifactorial cause of NEC [1]. Prematurity is the main risk factor, presumably due to immaturity of gastrointestinal motility, intestinal barrier function, and immune defence. Other contributing factors are thought to be genetic predisposition, enteral feeding, intestinal ischemia, and colonization with pathogenic bacteria [1, 4]. The condition is an inflammation in the intestines related to a harmful overreaction of the immature immune system to some insults [4]. The histological appearance shows bacterial invasion of the epithelia and early signs of necrosis of the enterocytes at the top of some villi [5].

Paneth cells are specialized epithelia that play a major role in the innate immune response [6]. They protect intestinal stem cells from pathogens, stimulate stem cell differentiation, and assist in repairing the intestine. The stem cell compartment is located at the base of the crypts, in the small intestine interspersed by Paneth cells [7]. Paneth cells act through the production of antimicrobial proteins/peptides [8]. The principal antimicrobial peptides, the alpha-defensins DEFA5 and DEFA6, differ in function [9]. DEFA6 shows weak activity against bacteria [10], and its antimicrobial function is activated by reducing milieu as a one-step mechanism by getting the more hydrophobic [11]. DEFA6 may have a key role in protecting the small intestine against invasion by diverse enteric pathogens through self-assembled peptide nanonets [12].

DEFA5 and DEFA6 mRNA levels are detectable at 13.5–17 weeks of gestation in the small intestine but with markedly diminished expression until the middle of the third trimester [13]. Enteric DEFA5 and DEFA6 mRNA levels are significantly lower in fetus and term newborns than in adults, and there are fewer numbers of Paneth cells in the crypts in extreme prematures than in term newborns and adults [13].

Recent studies suggest a key role for Paneth cells in the pathophysiology of NEC [5, 14–16]. The pathogenesis of NEC and inflammatory bowel Crohn's disease show many similarities [5, 17]. In Crohn's, the production of both DEFA5 and DEFA6 by Paneth cells is reduced [18]. Current knowledge of alpha-defensins in NEC is scanty, and the results seem to be controversial [19–21]. A better understanding of NEC is crucial to developing prevention and treatment strategies.

The aim of the study was to investigate the expression of DEFA6 in infants with NEC.

## 2. Materials and Methods

### 2.1. Study Population

Infants who underwent bowel resection for NEC at level III NICU in Sweden between August 2004 and September 2013 were eligible for the study (Table 1). The study protocol was approved by the Regional Ethical Review Board, and written informed consent was obtained from the parents.

Oral feeding with breast milk was started within two hours after birth in premature infants both in the control and the NEC groups. NEC was diagnosed by radiological and clinical features and staged according to the criteria of Bell et al. [22]. NEC diagnosis was confirmed during surgery and by histopathological evaluation. Infants with NEC were treated with broad-spectrum antibiotics, and enteral feeding was ceased prior to surgery. Infants with NEC underwent bowel resection, and an enterostomy was created. Those samples that represented macroscopically vital tissue, from ends of the resected intestine, were selected for further histopathological evaluation, and samples with complete mucosal erosion were excluded.

The controls all presented with delay to pass meconium, abdominal distention, and bilious vomiting, and they underwent laparotomy between four and 47 days of life and an ileostomy was created. Samples from the controls used for staining were taken from the site of the stoma. Three of the controls had transient functional immaturity of the intestine, and they were successfully managed with a temporary ileostomy (Table 1). Colonic biopsies in these three controls revealed the presence of immature ganglion cells, and ileal biopsies from the stoma showed the presence of normal ganglion cells. The fourth control was later diagnosed with pseudoobstruction and has still an ileostomy.

### 2.2. Intestinal Tissue Samples and Immunohistochemistry

We performed both the immunohistochemistry and the image analysis blindly. All samples were sectioned and stained on the same occasion for comparable analysis. Intestinal tissues were fixed in 4% formaldehyde in PBS for 24 h at 4°C. Embedded samples were sectioned (3 *μ*m) and mounted on SuperFrost slides. Samples were deparaffinized through a graded series of xylol-ethanol. To determine the Paneth cell-specific expression of DEFA6, immunohistochemistry was performed by the Benchmark Ultra system (Ventana) and the ultraView Universal DAB Detection Kit (Ventana) and even counterstaining with hematoxylin-eosin was carried out. Tissue sections were incubated with a polyconal rabbit anti-DEFA6 antibody (Prestige Antibodies® Powered by Atlas Antibodies, 1 : 800 dilution) after antigen retrieval in the CC2 buffer (Ventana) for 36 min. Negative control sections were prepared by performing immunostaining procedures without adding primary antibodies. Representative sections were digitally scanned with a 3DHISTECH scanner. The images were exported in TIFF format (10x magnification) with the virtual microscope software Panoramic Viewer (3DHISTECH).

### 2.3. Image Analysis

**Figure 1:**
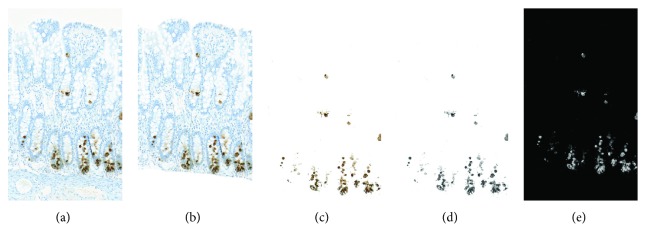
Image processing: DAB staining detection. (a) Original image—RGB color. (b) Selection of the mucosa as the region of interest (ROI). (c) IHC Toolbox plugin in ImageJ selects color, representing DAB staining. (d) Conversion of an RGB image to an 8-bit image (the lowest pixel intensity represents the highest DEFA6 expression and vice versa). (e) Inversion of pixel intensity values results in the highest pixel intensity corresponding to the highest DEFA6 expression.

ImageJ (freeware) was used for semiautomatic image analysis. The color detection of DAB staining was performed by the IHC Toolbox plugin in ImageJ, which can be effectively used to analyze samples stained by immunohistochemistry [23]. The model for color detection of brown pixels (which corresponded to the DAB staining and the level of DEFA6 expression) was adjusted specifically for the present project. The specificity of color detection was controlled visually. Working with image stacks during the evaluation process allowed the analysis to be made comparable between images. After color detection of DAB staining, the RGB color images were converted to 8-bit files. Inversion of the pixel intensity values resulted in higher pixel intensity corresponding to higher DEFA6 expression. The workflow is presented in Figures 1(a)–1(e). Before analysis, the same threshold window was set on each image in order to filter unspecific too high and/or too low pixel values. Besides the advantages of the method, even some limitations could be considered: the antigen retrieval process and the staining process are not easy to standardize and both can influence DAB intensity. Thus, the measured intensity levels in different studies are not directly comparable if they are performed at different time points.

### 2.4. DEFA6 Expression

Comparison of the general expression in the tissue was difficult since the height of the mucosa showed variations between patients. A solution was to measure the DAB-stained area as we hypothesized that the staining was homogeneous and had the same intensity for all the samples. We generated an even more standardized process, which showed DEFA6 expression for each *μ*m length of the mucosa. The mucosal part of the sections was selected manually as the region of interest (ROI) (Figure 1(b)), and the size of the area and the total DAB intensity of ROI were measured by ImageJ.

(1) $$\begin{array}{c} {}^{\text{“}}Length{}^{\text{”}}\text{ of }mucosa\text{ in the }ROI=\frac{Area\text{ of }ROI}{Mean\;height\text{ of }mucosa},\\ \frac{DEFA6\;expression}{\mu m}=\frac{Total\;DAB\;intensity\text{ of }ROI\;}{{}^{\text{“}}Length{}^{\text{”}}\text{ of }mucosa\text{ in the }ROI\;\left(\mu m\right)}. \end{array}$$

We calculated the mean value of the mucosal height (*μ*m) for each section from ten representative sites measured by the Panoramic Viewer software. Length in *μ*m could be converted into pixels and vice versa. The “length” of the mucosa part in the ROI is calculated by dividing the area in pixels with the height of the mucosa.

### 2.5. Statistics

Statistics were assessed in Microsoft Excel by two-sample *t*-test. A *P* value of <0.05 was considered to be statistically significant.

## 3. Results and Discussion

### 3.1. Study Population

A total of 12 infants were included in the study, eight with NEC and four controls. Patient characteristics are shown in Table 1. The tissue samples were taken from the colon (*n* = 1), jejunum (*n* = 1), and ileum (*n* = 10). Both the NEC and control groups consisted of extremely premature and term infants (control group: 25–40 gestational weeks, NEC group: 23–39 gestational weeks). The postnatal age at the time of surgery varied in both groups (control group: 4–47 days, NEC group: 4–50 days).

### 3.2. Tissue Characteristics and DEFA6 Expression

Searching in the database of the Human Protein Atlas revealed that DEFA6 is a highly specific marker for Paneth cells with exclusive expression in the intestine in adults [24].

The height of the mucosa varied highly in patients independently of the origin of the tissue or the indication for operation. No correlation between the height of the mucosa and the level of DEFA6 expression or gestational age could be seen (data not shown).

**Figure 2:**
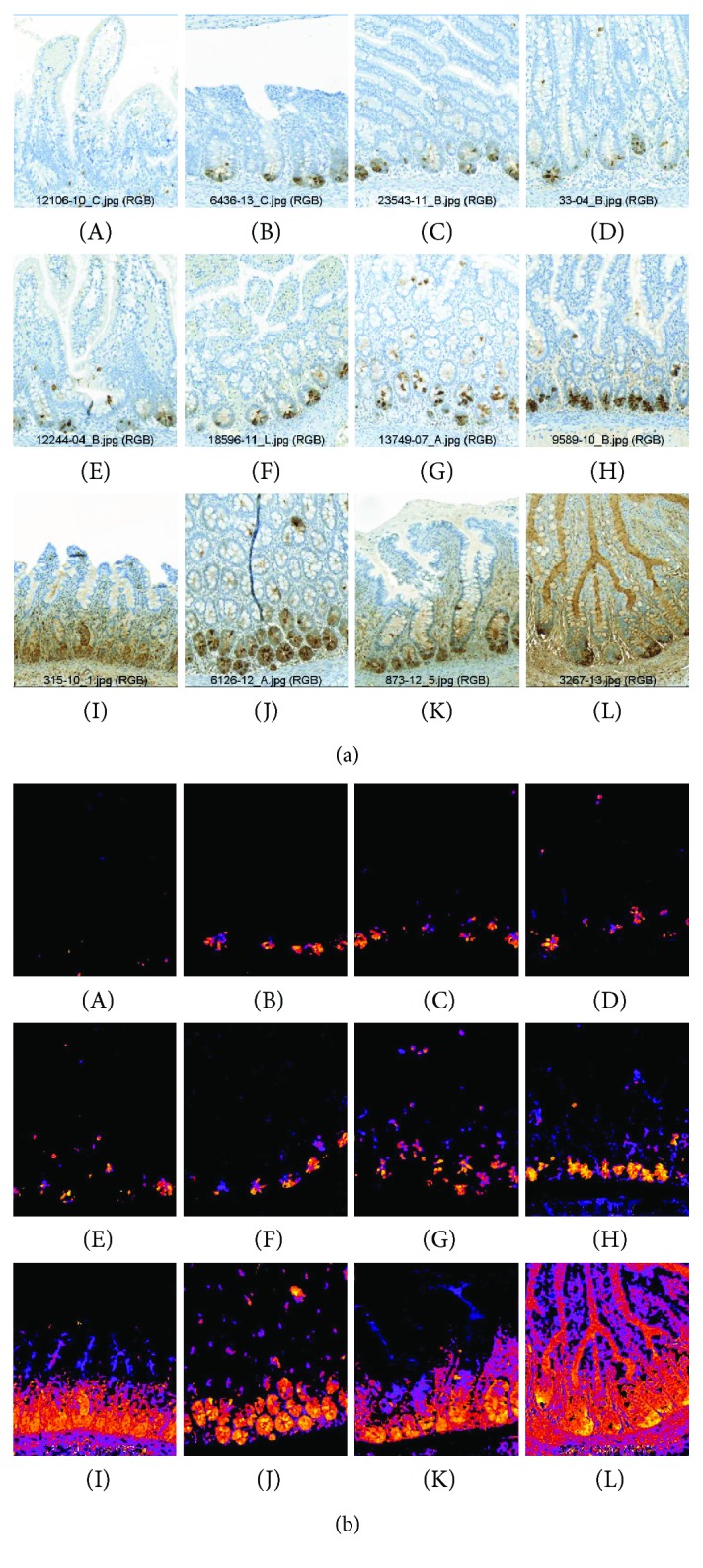
DEFA6 expression in the study material. (a) Increasing order of DEFA6 expression in patients. NEC group: A–F, H, and J; control group: G, I, K, L. (b) The lookup tables of images represent the distribution levels of DEFA6, visualized by colors (A–L). Extracellular DEFA6 expression appears in cases of higher general DEFA6 levels (H–L).

**Figure 3:**
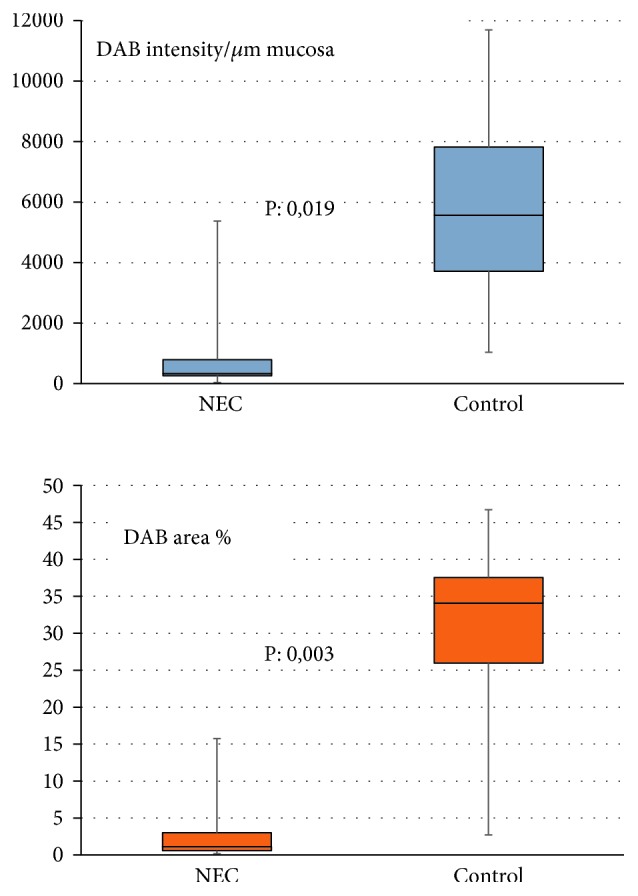
DEFA6 expression in the NEC group and the control group. Significant differences in DEFA6 expression irrespective of the evaluating method.

**Figure 4:**
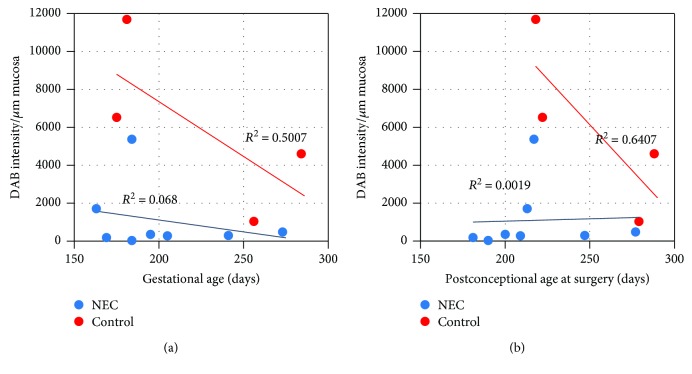
Relationship between DEFA6 expression and age. (a) Control patients showed higher level of DEFA6 expression and negative correlation between DEFA6 expression and gestational age. Premature controls had the highest DEFA6 level. Most of the NEC patients showed low DEFA6 expression. No correlation could be confirmed between DEFA6 expression and gestational age in the NEC group. (b) The phenomenon above was more pronounced between DEFA6 expression and postconceptional age at surgery (maturation level). corr: 0.03 (NEC group), corr: −0.74 (control group).

We found Paneth cells in all samples (Figure 2), even in the colon tissue (Figure 2, F). The tissue samples showed different levels of DEFA6 expression (Figure 3). Tissue samples may have varying water content that can influence DAB staining levels; therefore, we performed measurements on the DAB-covered area as well. DEFA6 expression was significantly lower in the NEC group than in the control group, independently of how we evaluated the DAB staining (DEFA6 expression/*μ*m mucosa (*P* = 0.019) or percent area of the mucosa with DEFA6 expression (*P* = 0.003)) (Figure 3). The DEFA6 expression in the NEC group remained low and did not correlate with gestational age; however, higher DEFA6 expression appeared in premature controls (Figure 4).

We were not able to estimate the objective count of Paneth cells due to the confluence of DAB staining in the sections with higher DEFA6 expression. However, the general impression suggests lower Paneth cell density in the NEC group (Figure 2).

### 3.3. Discussion

In the present study, we found a diminished DEFA6 expression in Paneth cells from infants with NEC compared to controls. Paneth cells and their main products, alpha-defensins, have been well studied in the development of Crohn's disease in adults [7, 16]. In Crohn's, the production of DEFA5 and DEFA6 by Paneth cells was reduced [15], which results in a defective antimicrobial shield and dysfunction of the mucosal barrier [9]. In premature infants, the diminished expression of DEFA5 and DEFA6 may result in higher risk of infection and inflammation. Although recent studies have suggested a Paneth cell dysfunction in NEC [5, 11–13], studies in alpha-defensin DEFA5 and DEFA6 expression in NEC patients are restricted to two previous reports [20, 21]. They investigated the mRNA levels of DEFA5 and DEFA6 and protein level of only DEFA5 but not DEFA6 [20, 21]. An explanation to the absence of studies in DEFA6 protein levels in NEC might be that the reliable commercial antibody for immunohistochemistry was not previously available. The use of immunohistochemistry in our study enabled structural evaluations. The disadvantage of this method is that it detects the actual protein levels, which may not correlate with mRNA levels in case of circumstances with enhanced protein degradation.

Salzman et al. found elevated DEFA5 and DEFA6 mRNA levels in infants with NEC compared with five near-term controls (four patients with intestinal atresia and one with meconium ileus) [20]. Intracellular peptide levels in NEC did not coincide with the elevation in mRNA. Puiman et al. found decreased DEFA5 protein expression in NEC patients [21]. The control group consisted of various diagnoses, namely, intestinal atresia, volvulus, gastroschisis, cloacal malformation, milk curd obstruction, Meckel's diverticulum, and ileus. The comparison with results of other studies is complicated by methodological heterogeneity and variation in gestational age between study populations and variation in diagnosis in the control groups [20, 21]. Nevertheless, similar to our results, alteration in the expression of alpha-defensins DEFA5 and DEFA6 seems to be associated with NEC [20, 21].

The number of Paneth cells [25] and the enteric expression of alpha-defensins in the fetus are low [13]; however, the production after birth is inducible by multiple factors. Both intraluminal bacteria and lipopolysaccharide can stimulate Paneth cell secretion, as well as cholinergic agonists and feeding [25–28]. This corresponds to our observation that DEFA6 expression was high in our fed extremely premature controls. An interpretation of our observations is that the DEFA6 expression is initially low at birth, inducible, and increasing in healthy individuals but remains low in patients who develop NEC.

Interestingly, we found Paneth cell metaplasia in a colonic tissue sample with NEC. This phenomenon has been previously reported in inflammatory bowel disease [29]. Puiman et al. described metaplastic Paneth cells in post-NEC stricture colon samples but not in NEC samples. They suggested that chronic inflammation caused Paneth cell metaplasia [21].

The strength of this study was that it was one of the two studies in DEFA6 expression and NEC. Moreover, we described the setup of a reliable semiquantitative method to study the expression of DEFA6.

The limitations of our study were the small sample size and the fact that the numbers of age-matched control patients were limited since material collection from a healthy control group was not feasible due to ethical reasons. The same problem appeared in previous studies of DEFA5 and DEFA6 where patients with various diagnoses such as volvulus, gastroschisis, small intestinal atresia, and ileus were included as controls [20, 21].

## 4. Conclusions

The diminished DEFA6 expression in Paneth cells associated with NEC in this study supports the hypothesis that alpha-defensins are involved in the pathophysiology of NEC. Future studies are needed to elucidate the role of alpha-defensins in NEC aiming at finding preventive and therapeutic strategies against NEC.

## Figures and Tables

**Table 1:** Study population.

Patient code	Diagnosis	Tissue origin	Gestational age at birth (weeks + days)	Birth weight (grams)	Gestational age at surgery (weeks + days)	Postnatal age at surgery (days)	Gender
12244-04_B1	NEC	Ileum	27 + 6	1438	28 + 4	5	Male
9589-10_B	NEC	Ileum	23 + 2	585	30 + 3	50	Male
12106-10_C	NEC	Ileum	26 + 2	1025	27 + 1	6	Male
18596-11_L	NEC	Colon	39 + 0	2360	39 + 4	4	Male
23543-11_B	NEC	Ileum	29 + 2	1225	29 + 6	4	Male
6126-12_A	NEC	Jejunum	26 + 2	912	31 + 0	33	Male
6436-13_C	NEC	Ileum	24 + 1	597	25 + 6	12	Female
33-04_B	NEC	Ileum	34 + 3	2300	35 + 2	6	Female
13749-07_A	Dysmotility	Ileum	36 + 4	3075	39 + 6	23	Male
3267-13	Dysmotility	Ileum	25 + 6	622	31 + 1	37	Female
315-10_1	Dysmotility	Ileum	40 + 4	4320	41 + 1	4	Female
873-12_5	Dysmotility	Ileum	25 + 0	870	31 + 5	47	Female

## Data Availability

The data used to support the findings of this study are included within the article.
